# Pulmonary Hypertension Is Associated with Worse Outcomes in Patients Hospitalized for Sick Sinus Syndrome

**DOI:** 10.19102/icrm.2023.14105

**Published:** 2023-10-15

**Authors:** Richard Orji, Favour Markson, Ayodeji Ilelaboye, Emeka Okoronkwo, Hafeez Shaka, Hakeem Ayinde, Tonye Teme

**Affiliations:** 1 Department of Medicine, Rosalind Franklin University of Medicine & Science, North Chicago, IL, USA; 2 Department of Medicine, Northwestern Medicine McHenry Hospital, McHenry, IL, USA; 3 Department of Management, University of Massachusetts Amherst, Amherst, MA, USA; 4 Department of Medicine, Lincoln Medical Center, New York, NY, USA; 5 Department of Medicine, Lautech Teaching Hospital, Ogbomoso, Oyo, Nigeria; 6 Department of Medicine, Lagos University Teaching Hospital, Lagos, Nigeria; 7 Department of Medicine, John H. Stroger, Jr. Hospital of Cook County, Chicago, IL, USA; 8 Division of Cardiology, Novant Health Heart & Vascular Institute Charlotte, Charlotte, NC, USA; 9 Division of Cardiology, Northwestern Medicine, McHenry Hospital, McHenry, IL, USA

**Keywords:** Cardiac arrest, cardiogenic shock, pacemaker insertion, pulmonary hypertension, sick sinus syndrome

## Abstract

Sick sinus syndrome (SSS) is a condition of the sinoatrial node that arises from a constellation of aberrant rhythms, resulting in reduced pacemaker activity and impulse transmission. According to the World Health Organization, pulmonary hypertension (PH) is defined by a mean pulmonary arterial pressure of >25 mmHg at rest, measured during right heart catheterization. It can result in right atrial remodeling, which may predispose the patient to sinus node dysfunction. This study sought to estimate the impact of PH on clinical outcomes of hospitalizations with SSS. The U.S. National Inpatient Sample database from 2016–2019 was searched for hospitalized adult patients with SSS as a principal diagnosis with and without PH as a secondary diagnosis using the International Classification of Diseases, Tenth Revision, codes. The primary outcome was inpatient mortality. The secondary outcomes were acute kidney injury (AKI), cardiogenic shock (CS), cardiac arrest, rates of pacemaker insertion, total hospital charges (THCs), and length of stay (LOS). Multivariate regression analysis was used to adjust for confounders. A total of 181,230 patients were admitted for SSS; 8.3% (14,990) had underlying PH. Compared to patients without PH, patients admitted with coexisting PH had a statistically significant increase in mortality (95% confidence interval, 1.21–2.32; *P* = .002), AKI (*P* < .001), CS (*P* = .004), THC (*P* = .037), and LOS (*P* < .001). In conclusion, patients admitted primarily for SSS with coexisting PH had a statistically significant increase in mortality, AKI, CS, THC, and LOS. Additional studies geared at identifying and addressing the underlying etiologies for PH in this population may be beneficial in the management of this patient group.

## Introduction

Sick sinus syndrome (SSS), also known as sinus node dysfunction, is a heart rhythm disorder accompanied by diverse symptoms such as fatigue, chest discomfort, syncope, palpitations, shortness of breath on exertion, dizziness, and hypotension.^1,2^ In the United States, SSS occurs in about 1 in 600 cardiac patients aged >65 years, making it one of the most common indications for pacemaker insertion (PMI) nationwide.^3^ A study reports SSS as the primary indication for PMI in approximately 30%–50% of U.S. cases.^4^

Studies have assessed predictors of poor outcomes in sinus nodal disease^5,6^; Greenspon et al. noted in their research that comorbidities such as hypertension, a prior history of systemic embolism, previous stroke or transient ischemic attack, prior systemic embolism, and heart failure with a New York Heart Association functional class of III or IV were predictive of a poor outcome.^7^

According to the World Health Organization (WHO), pulmonary hypertension (PH) is defined by a mean pulmonary arterial pressure of >25 mmHg at rest, measured during right heart catheterization.^8^ Research has shown an increased incidence of arrhythmia in patients with PH.^9^ About 15%–20% of patients with WHO class III and IV PH have been shown to have one or more forms of arrhythmia, including sinus node dysfunction.^10,11^ The prolonged elevation of atrial pressure in patients with PH induces progressive electrophysiological remodeling which, in conjunction with autonomic system modulations, may predispose these patients to the development of atrial arrhythmias.^12^ Additionally, 17% of deaths in PH are sudden and unexpected, many of which are due to arrhythmia.^13^

The aim of our study was to assess the clinical outcomes of SSS in patients hospitalized with coexisting PH and also to buttress the need for delicate care and management in this patient population.

## Materials and methods

Data were sourced from the U.S. Nationwide Inpatient Sample (NIS) database from 2016–2019. The NIS is a hospital inpatient stay database derived from hospitals’ billing data to statewide data organizations across the United States, covering >97% of the U.S. population.^14^ Each year approximates a 20% stratified sample of discharges from U.S. community hospitals, excluding rehabilitation and long-term acute care hospitals. This dataset is weighted to obtain national estimates. The 2016–2019 datasets are coded using the International Classification of Diseases, Tenth Revision, Clinical Modification/Procedure Coding System (ICD-10-CM/PCS). In the NIS, diagnoses are divided into a principal diagnosis and a secondary diagnosis. For our purposes, a principal diagnosis was the main ICD-10 code for the hospitalization; secondary diagnoses were any ICD-10 code other than the principal diagnosis.

We conducted a retrospective cohort study of hospitalizations from 2016–2019 with a principal diagnosis of SSS and a secondary diagnosis of PH. We used ICD-10 codes obtained from a literature review of similar validated studies on SSS.^15^

This study was exempt from Northwestern Medicine Institutional Review Board approval as it involves data without patient identifiers. The data used in this study are readily available online at www.hcup-us.ahrq.gov.

**Figure 1:**
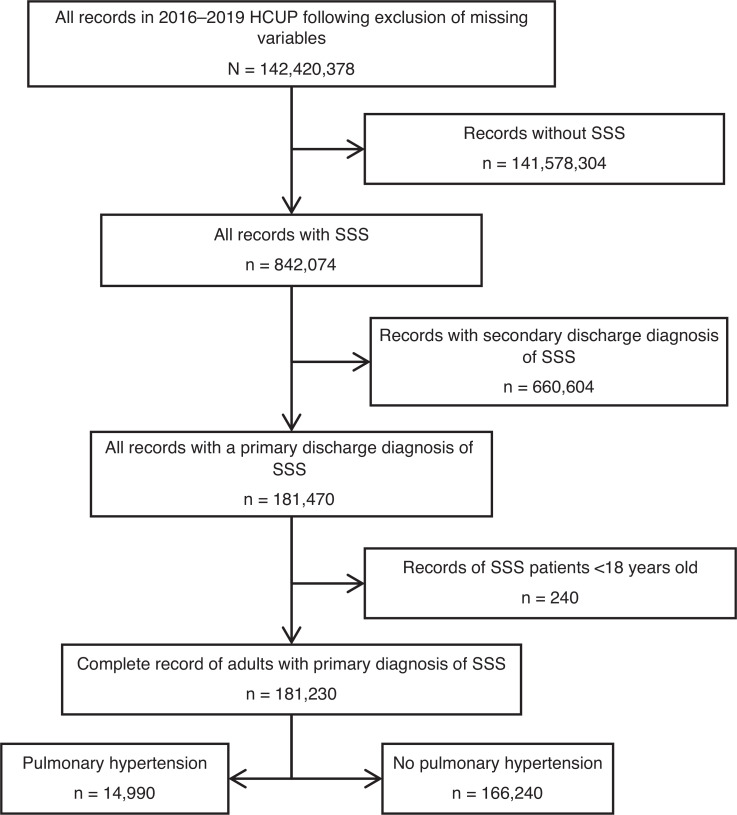
Selection flowchart of patients from the Healthcare Cost and Utilization Project database. *Abbreviations:* HCUP, Healthcare Cost and Utilization Project; n, number of patients; SSS, sick sinus syndrome.

The study population consisted of principal hospitalizations for SSS in the NIS from 2016–2019. Study variables included sociodemographic characteristics, medical comorbidities, hospital characteristics, and primary and secondary outcomes (mentioned later). ICD-10 codes were used to identify the principal and secondary diagnoses **(Supplementary Table 1)**. We excluded patients aged <18 years. Baseline hospitalization characteristics for SSS with and without a secondary diagnosis of PH were studied (selection flowchart in **Figure 1**).

The primary outcome compared inpatient mortality for SSS with and without a secondary diagnosis of PH. Secondary outcomes included rates of complications like acute kidney injury (AKI), cardiogenic shock (CS), and cardiac arrest (CA) and rates of PMI. We also compared the mean length of stay (LOS) and the mean total hospital charges (THCs) for SSS with and without a secondary diagnosis of PH as a surrogate marker for health care cost utilization. We analyzed the data using STATA version 17 (StataCorp, College Station, TX, USA). We conducted all the analyses using the weighted samples for national estimates, following Healthcare Cost and Utilization Project (HCUP) regulations for using the NIS database.

A univariate logistic regression analysis using all variables and comorbidities presented in **Table 1** was used to calculate unadjusted odds ratios for the outcomes of SSS hospitalizations with or without a secondary diagnosis of PH. Variables and comorbidities were selected from the literature review. All variables with *P* < .1 were included in a multivariate logistic regression model, which was used to calculate the adjusted odds ratios (aORs) for our study outcomes. The final model consisted of age, sex, race, atrial fibrillation, chronic heart failure, chronic obstructive pulmonary disease (COPD), oxygen dependence, the Elixhauser Comorbidity Index, anemia, diabetes mellitus, hypothyroidism, carotid artery disease, anemia, liver disease, chronic kidney disease, electrolyte derangements, and maintenance dialysis. *P* values considered significant in the multivariate analysis were two-sided, with .05 as the threshold for statistical significance. A univariate analysis for outcomes of SSS in patients with coexisting PH is shown in **Table 2**.

## Results

There were 181,230 hospitalizations for SSS, of which 14,990 (8.3%) had a secondary diagnosis of PH. Patients with PH were significantly older (mean age, 79.2 vs. 76.5 years; *P* < .001) and included a lower proportion of men compared to patients without PH. SSS hospitalizations with coexisting PH encompassed a greater proportion of non-White patients than those without PH.

The comorbidity distribution between both cohorts varied. Patients with PH included higher proportions of those COPD (26.3% vs. 15.2%, *P* < .001), chronic heart failure (58.3% vs. 26.5%, *P* < .001), peripheral vascular disease (6.5% vs. 4.8%, *P* < .001), atrial fibrillation (76.9% vs. 57.6%, *P* < .001), hypothyroidism (26.8% vs. 21.3%, *P* < .001), and oxygen dependence (6.9% vs. 2.2%, *P* < .001). There was also significant racial distribution relative to PH, as shown in **Table 1**.

**Figure 2:**
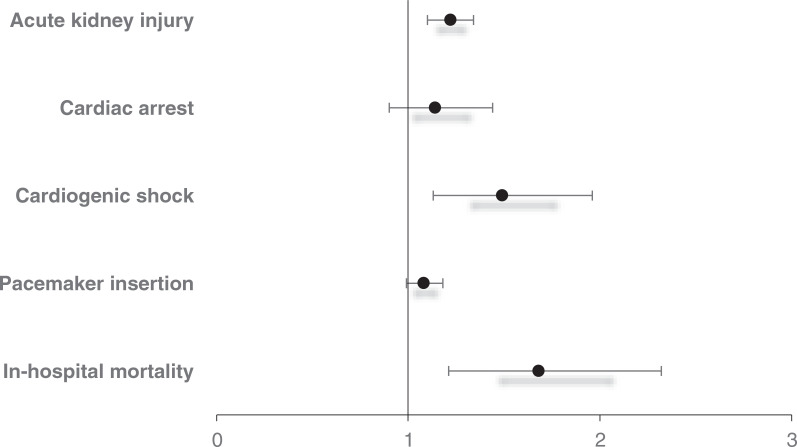
Forest plot for in-hospital outcomes of patients hospitalized with sick sinus syndrome and coexisting pulmonary hypertension. Effect measures are presented as adjusted odds ratios.

The in-hospital mortality for patients admitted for SSS with coexisting PH was 1.84% compared to 0.59% for patients without PH (aOR, 1.68; 95% confidence interval [CI], 1.21–2.32; *P* = .002) when adjusted for biodemographic and hospital characteristics as well as comorbidities **(Figure 2)**.

Patients admitted with SSS and coexisting PH had increased odds (of 22% and 49%, respectively) for AKI and CS compared to patients without PH. However, there was no difference in the rates of PMI and CA between the two groups. Patients with SSS and coexisting PH had a higher mean LOS and mean THC when compared to patients without PH, as detailed in **Table 3**.

## Discussion

In this study, patients with SSS coexisting with PH had significantly higher in-hospital mortality rates, which may be related to the increased risk for electrolyte derangements and acute right ventricular failure (right ventricular hypertrophy) resulting from PH. PH is one of the major causes of acute right-sided heart failure and is often fatal in this population.^16^ PH is associated with electrolyte derangement and hyponatremia. Low serum sodium is a sequela of fluid retention resulting from heart failure in PH.^17^ These may explain the increased mortality in patients with SSS and coexisting PH. Also, the increased odds of developing CS and AKI discovered in this study can explain the increased in-hospital mortality in these patients. In their study, D’Alonzo et al. noted that right ventricular hemodynamic dysfunction was closely linked to increased mortality.^18^

We also noted that patients with coexistent PH had significantly longer hospital stays. This may be attributed to the attendant pulmonary, cardiac, and vascular complications associated with PH. Also, the hemodynamic and electrolyte disturbances seen in PH may explain the longer hospital stay. Campo et al. noted that right ventricular dysfunction and electrolyte imbalances contribute to prolonged hospitalization and morbidity in arrhythmogenic PH.^19^ Haddad et al. identified renal dysfunction on admission, hyponatremia, and tricuspid regurgitation as independent risk factors for prolonged hospitalization.^20^

Our study revealed that SSS patients with coexisting PH had significantly higher hospitalization costs, which could be associated with an extended hospital stay. Also, the emergence of other complications during a prolonged hospital stay, such as pulmonary thromboembolism and AKI, may inevitably lead to the increased need for interventions and medications. Anand et al. noted that, although novel therapies correlate with significantly decreased PH-related hospitalizations in the United States, hospital charges have risen substantially and are increasingly being borne by Medicare.^21^

Our study noted that the odds of AKI were higher in patients with SSS coexisting with PH. Probable mechanisms for this relationship include increased venous congestion, reduced cardiac output, renin–angiotensin–aldosterone system activation, and the presence of circulating cytokines that could potentially worsen kidney function. All these mechanisms can occur due to right ventricular dysfunction, a sequela of PH, and impaired cardiac output compounded by SSS.^22,23^ Kidney injury that results from PH has been associated with an increase in mortality, further explaining the increased mortality in patients with both SSS and PH.^24^

Patients with coexisting PH experienced higher rates of cardiovascular events such as CS, which can be attributed to right ventricular dysfunction. Right ventricular dysfunction/failure has also been shown to cause left ventricular dysfunction. Increased pulmonary vascular resistance in PH triggers a reduction in the right ventricular stroke volume, which may result in the bowing of the left ventricular septum and impairment of left ventricular filling. According to the Frank–Starling law, the decrease in left ventricular preload reduces stroke volume, thereby affecting the cardiac output and ultimately resulting in CS (cardiac output = heart rate × stroke volume).^25^ Dong et al. noted that right ventricular dysfunction was one of the most important prognostic factors for major cardiovascular events in PH.^26^

Our study has several strengths. It is one of the few studies evaluating SSS outcomes in patients with coexisting PH. Using the largest inpatient hospitalization database in the United States increases the power of our study. Our scientific questioning and analysis technique also contributes new information to a largely understudied topic of outcomes of SSS in patients with coexisting PH.

### Study limitations

The database used for this study (NIS), despite containing information on individual hospitalization, does not account for the complex nature of hospitalizations. Also, there may be some residual effects of sampling errors and clustering and stratification biases. The retrospective nature of our study establishes associations but cannot imply causality. Our study had more SSS patients without PH than patients with SSS and PH. The NIS reports data on hospitalizations rather than individual patients; hence, patients hospitalized on multiple occasions can be counted multiple times.^27^ Finally, the NIS does not account for the acuity or severity of PH, so we could not determine whether this may have affected our hospital outcomes.^28^

## Conclusion

Hospitalizations for SSS with coexisting PH had a statistically significant increase in mortality, AKI, CS, THC, and LOS compared to those without PH. Identifying and addressing the underlying etiologies for PH in this population may improve clinical outcomes. It is also essential for clinicians to consider dual-chamber pacemakers, especially in cohorts with PH, to minimize the risk of pacemaker syndrome. We encourage further studies to analyze and evaluate this association.

## Figures and Tables

**Table 1:** Patient and Hospital Characteristics for Hospitalizations with Sick Sinus Syndrome and Coexisting Pulmonary Hypertension

Variable	PH, %	No PH, %	*P* Value
n = 181,230	n = 14,990 (8.3%)	n = 166,240 (91.7%)	
Patient age (years), mean ± SE	79.2 ± 1.8	76.5 ± 0.6	<.001
Patient sex			<.001
Male	35.8	47.9	
Female	64.2	52.1	
Racial distribution			.009
White	78.6	79.5	
Black	9.6	7.7	
Hispanic	6.3	7.0	
Asian	3.0	3.3	
Native American	0.5	0.4	
Other	2.0	2.1	
Hospital region			<.001
Northeast	18.7	18.3	
Midwest	24.6	21.6	
South	35.8	40.2	
West	20.9	19.9	
Hospital bed size			.289
Small	16.5	17.5	
Medium	31.1	31.5	
Large	52.4	51.0	
Hospital teaching status			<.001
Nonteaching hospital	28.6	32.8	
Teaching hospital	71.4	67.2	
Hospital location			.002
Rural	5.7	7.3	
Urban	94.3	92.7	
Elixhauser Comorbidity Index score (points)			<.001
1	4.3	0	
2	13.9	0.6	
≥3	81.8	99.4	
Primary payer			<.001
Medicare	83.9	89.4	
Medicaid	3.7	2.9	
Private	11.6	7.1	
Uninsured	0.9	0.6	
Median annual income in patient’s zip code (USD)^a^			.362
$1–$43,999	26.8	26.8	
$44,000–$55,999	27.0	27.0	
$56,000–$73,999	24.7	24.7	
≥$74,000	21.5	21.5	
Comorbidities			
Dyslipidemia	60.9	59.2	.074
History of MI	11.2	9.5	.002
History of PCI	0.9	1.2	.198
History of CABG	10.9	10.3	.319
Atrial fibrillation	76.9	57.6	<.001
COPD	26.3	15.2	<.001
Carotid arterial disease	3.9	3.1	.011
History of stroke	0.7	0.9	.310
Peripheral vascular disease	6.5	4.8	.001
Hypothyroidism	26.8	21.3	<.001
Diabetics mellitus	37.3	31.2	<.001
Chronic kidney disease	42.4	25.2	<.001
Chronic heart failure	58.3	26.5	<.001
Smoking	28.5	28.0	.567
Liver disease	3.3	2.2	.001
Electrolyte derangement	29.6	18.7	<.001
Maintenance dialysis	4.6	1.8	<.001
Oxygen dependence	6.9	2.2	<.001
Anemia	34.0	18.5	<.001
Obesity	18.9	13.5	<.001

*Abbreviations:* CABG, coronary artery bypass graft; COPD, chronic obstructive pulmonary disease; MI, myocardial infarction; PCI, percutaneous coronary intervention; PH, pulmonary hypertension; SE, standard error; SSS, sick sinus syndrome; USD, United States dollars. ^a^Median annual income in patient’s zip code from 2016–2019.

**Table 2:** Univariate Results for Outcomes of Sick Sinus Syndrome in Patients with Coexisting Pulmonary Hypertension

Outcome	aOR (95% CI)	*P* Value
Primary outcome		
In-hospital mortality	3.15 (2.34–4.24)	<.001
Secondary outcomes		
Pacemaker insertion	0.90 (0.83–0.98)	.015
Cardiogenic shock	2.68 (2.09–3.45)	<.001
Cardiac arrest	1.10 (0.88–1.38)	.398
Acute kidney injury	2.01 (1.89–2.24)	<.001
	**aMR (95% CI)**	***P* Value**
Length of hospital stay	1.27 (1.12–1.42)	<.001
Total hospital charges (USD)	$11,426 ($8649–14,203)	<.001

*Abbreviations:* aOR, adjusted odds ratio; aMR, adjusted mean ratio; CI, confidence interval; PH, pulmonary hypertension; SSS, sick sinus syndrome; USD, United States dollars.

**Table 3:** Multivariate Results for Outcomes of Sick Sinus Syndrome in Patients with Coexisting Pulmonary Hypertension

Outcome	PH, %	No PH, %	aOR (95% CI)	*P* Value
n = 181,230	n = 14,990 (8.3%)	n = 166,240 (91.7%)		
Primary outcome				
In-hospital mortality	1.84	0.59	1.68 (1.21–2.32)	.002
Secondary outcomes				
Pacemaker insertion	73.0	75.1	1.08 (0.99–1.18)	.104
Cardiogenic shock	2.6	1.0	1.49 (1.13–1.96)	.004
Cardiac arrest	2.9	2.6	1.14 (0.90–1.44)	.277
Acute kidney injury	28.8	16.5	1.22 (1.10–1.34)	<.001
			**aIRR (95% CI)**	***P* Value**
Length of hospital stay	5.2	4.0	1.09 (1.06–1.12)	<.001
Total hospital charges (USD)	$90,240.5	$78,813.9	1.03 (1.01–1.06)	.037

*Abbreviations:* aOR, adjusted odds ratio; aIRR, adjusted incidence rate ratio; CI, confidence interval; PH, pulmonary hypertension; SSS, sick sinus syndrome; USD, United States dollars.

**Supplementary Table 1:** ICD-10 Codes of Study Variables

	ICD-10 Codes
Diagnosis codes	
Pulmonary hypertension	I270, I272
Sick sinus syndrome	I495
Outcome variables	
Acute kidney injury	N17
Cardiac arrest	I46
Cardiogenic shock	R570
Pacemaker insertion	OJH604Z, OJH605Z, OJH606Z
Comorbidities	
Atrial fibrillation	I480, I481, I482, I4891
Anemia	D50, D51, D52, D53, D55, D56, D57, D58, D59, D60, D61, D62, D63, D64
Carotid arterial disease	I652
Chronic heart failure	I50
Chronic kidney disease	N18
COPD	J41, J42, J43, J44
Diabetics mellitus	E11
Dyslipidemia	E78
Electrolyte derangement	E870, E871, E872, E873, E874, E875, E876
History of CABG	Z951
History of MI	I252
History of PCI	Z9861
History of stroke	I63
Hypothyroidism	E03
Liver disease	K70, K71, K72, K73, K74, K75, K76, K77
Maintenance dialysis	Z992
Obesity	E660, E6601, E6609, E661, E662, E668, E669
Oxygen dependence	Z9981
Peripheral vascular disease	I739
Smoking	Z87891, F17200

*Abbreviations:* CABG, coronary artery bypass graft; COPD, chronic obstructive pulmonary disease; ICD-10, International Classification of Diseases, Tenth Revision; MI, myocardial infarction; PCI, percutaneous coronary intervention.
